# Genomic Selection for Cashmere Traits in Inner Mongolian Cashmere Goats Using Random Forest, Gradient Boosting Decision Tree, Extreme Gradient Boosting and Light Gradient Boosting Machine Methods

**DOI:** 10.3390/ani15202940

**Published:** 2025-10-10

**Authors:** Jiaqi Liu, Xiaochun Yan, Wenze Li, Shan-Hui Xue, Zhiying Wang, Rui Su

**Affiliations:** 1 College of Animal Science, Inner Mongolia Agricultural University, Hohhot 010018, China; eliauk_1371295874@163.com (J.L.);; 2 Sino-Arabian Joint Laboratory of Sheep and Goat Germplasm Innovation, Hohhot 010018, China; 3 Inner Mongolia Key Laboratory of Sheep & Goat Genetics Breeding and Reproduction, Hohhot 010018, China

**Keywords:** genomic selection, Inner Mongolian cashmere goats, machine learning

## Abstract

**Simple Summary:**

This study aims to perform genome selection of cashmere traits in Inner Mongolian cashmere goats using machine learning algorithms. By comparing the prediction accuracy of various machine learning algorithms, it explores the feasibility of applying different machine learning algorithms to genome selection of cashmere traits in Inner Mongolian cashmere goats, with the goal of improving the accuracy of genomic selection and enhancing breeding efficiency. Fiber length and cashmere production can enhance the economic value of cashmere goats. We analyzed cashmere trait data from 2299 cashmere goats, including fiber length, cashmere diameter, and cashmere production. We used RF, XGBoost, GBDT, and LightGBM for genome selection in Inner Mongolian cashmere goats. For fiber length, cashmere production, and cashmere diameter, LightGBM, RF, and GBDT achieved the highest selection accuracy after hyperparameter optimization. However, in the case of cashmere traits, the prediction accuracy of XGBoost was the lowest among all the models, at 0.541, 0.309, and 0.387 for fiber length, cashmere production, and cashmere diameter, respectively. For machine learning methods, hyperparameter tuning is essential, as it can improve prediction accuracy.

**Abstract:**

In recent years, Machine Learning (ML) has garnered increasing attention for its applications in genomic prediction. ML effectively processes high-dimensional genomic data and establishes nonlinear models. Compared to traditional Genomic Selection (GS) methods, ML algorithms enhance computational efficiency and offer higher prediction accuracy. Therefore, this study strives to achieve the optimal machine learning algorithm for genome-wide selection of cashmere traits in Inner Mongolian cashmere goats. This study compared the genomic prediction accuracy of cashmere traits using four machine learning algorithms—Random Forest (RF), Extreme Gradient Boosting Tree (XGBoost), Gradient Boosting Decision Tree (GBDT), and LightGBM—based on genotype data and cashmere trait phenotypic data from 2299 Inner Mongolian cashmere goats. The results showed that after parameter optimization, LightGBM achieved the highest selection accuracy for fiber length (56.4%), RF achieved the highest selection accuracy for cashmere production (35.2%), and GBDT achieved the highest selection accuracy for cashmere diameter (40.4%), compared with GBLUP, the accuracy improved by 0.8–2.7%. Among the three traits, XGBoost exhibited the lowest prediction accuracy, at 0.541, 0.309, and 0.387. Additionally, following parameter optimization, the prediction accuracy of the four machine learning methods for cashmere fineness, cashmere yield, and fiber length improved by an average of 2.9%, 2.7%, and 3.8%, respectively. The mean squared error (MSE) and mean absolute error (MAE) for all machine learning methods also decreased, indicating that hyperparameter tuning can enhance prediction accuracy in ML algorithms.

## 1. Introduction

Inner Mongolian cashmere goats are a superior local breed of China developed through long-term natural selection and artificial breeding, the cashmere produced is soft, white, and highly elastic, and the goats are renowned for their high cashmere production and excellent quality [1,2]. The Inner Mongolian cashmere goat population is primarily concentrated in the western regions of the Inner Mongolia Autonomous Region, categorized by origin into Albasi, Eriliangshan, and Alxa types. Among them, the cashmere from the Albasi type is renowned for its exceptional quality, earning it the nicknames ‘soft gold’ and ‘fiber gem’ [3]. In an effort to improve the breeding efficiency of fiber-related traits in Inner Mongolian cashmere goats, genomic selection has been applied.

Genomic selection (GS) (also known as genomic prediction, CP) uses genome-wide molecular markers and individual phenotypic data to construct a mixed model for genetic evaluation of individuals. Since Meuwissen et al. (2001) published their groundbreaking research on predicting breeding values using genome-wide single-nucleotide polymorphisms (SNP) [4], the concept of “genomic selection” has revolutionized the field of plant and animal breeding. To date, several approaches have been proposed to develop more efficient statistical methods for estimating genomic estimated breeding values (GEBV), such as the widely used genomic best linear unbiased prediction (GBLUP) in GS [5], single-step BLUP (ssGBLUP) [6], ridge regression, Bayesian regression [4], and emerging machine learning (ML) strategies, including support vector regression (SVR) [7], random forests (RF) [8], reproducing kernel Hilbert space regression (RKHS), and kernel ridge regression. According to Howard et al. [9], single-step genomic best linear unbiased prediction (ssGBLUP) exhibited higher accuracy compared to BLUP. Zhang et al. [10] implemented ABLUP, GBLUP, and ssGBLUP methods to perform genomic selection for early growth traits in Inner Mongolian cashmere goats. ssGBLUP exhibited higher prediction accuracy compared to GBLUP and ABLUP. Negro et al. [11] used BLUP, GBLUP, and ssGBLUP methods to perform genomic selection for milk-related traits in Saanen and Alpine goats. The results showed that compared to BLUP, GBLUP and ssGBLUP had higher prediction accuracy and correlation.

Machine learning (ML), also termed automated knowledge acquisition, is an efficient data-processing methodology and has been extensively utilized in the field of animal genetics and breeding [12,13,14]. Machine Learning efficiently handles high-dimensional genomic data and constructs nonlinear models, allowing for more accurate prediction of animal genetic value [15]. In ML, the three most widely used learning methods are: supervised learning, unsupervised learning, and reinforcement learning. Compared with traditional genomic selection methods, machine learning algorithms demonstrate superiority in computational efficiency and prediction accuracy. The computational efficiency of the entire process is further improved through optimization algorithms in machine learning. Cross-validation is utilized during model selection to make optimal use of sample information and enhance prediction accuracy. LIANG et al. [16] evaluated the accuracy of five genomic selection methods (SVR, KRR, RF, Adaboost. RT, and GBLUP) in Chinese Simmental beef cattle. Results indicated that the four machine learning methods improved accuracy by 12.8%, 14.9%, 5.4%, and 14.4% over the traditional GBLUP method. Xiang et al. [17] compared the performance of linear genomic selection models and nonlinear machine learning models for genomic prediction of pig production traits. The results demonstrated that machine learning algorithms outperformed linear genomic selection models in terms of prediction accuracy for pig production traits. Among all machine learning algorithms, support vector machines (SVMs) exhibited the highest genomic prediction accuracy.

This study employed Random Forest (RF), XGBoost, Gradient Boosting Decision Tree (GBDT), and LightGBM to perform genomic selection for cashmere traits in Inner Mongolian cashmere goats, compared the genomic prediction accuracies of these machine learning methods for cashmere traits, investigated the feasibility of applying different genomic selection strategies to cashmere traits of Inner Mongolian cashmere goats, with the aim of improving genomic prediction accuracy and accelerating genetic improvement within the population.

## 2. Materials and Methods

### 2.1. Experimental Materials

Phenotypic Data: The phenotypic data used in this study were sourced from Inner Mongolian Cashmere Goats from the Inner Mongolia Yiwei White Cashmere Goat Co., Ltd., Ordos, China. The traits selected for analysis included fiber length, cashmere production, and fiber diameter. The goat farm employed a rotational herding management approach to ensure scientific feeding and care for the goats. In October of each year, the farm conducted synchronized breeding using artificial insemination, with detailed records of breeding data to ensure precise control of the reproduction process. Cashmere lambs were born in March, and pedigree data were recorded. The shearing season occurred annually between April and May, during which data on fiber length, cashmere production, and other traits were recorded. Fiber length was measured by shaving a 10 cm^2^ patch on the goat’s scapular area before shearing. Samples of cashmere were sent to the Cashmere Analysis Laboratory at Inner Mongolia Agricultural University. The samples underwent separation of fiber and cashmere, followed by cleaning with petroleum ether to remove debris such as soil and grease. Finally, the fiber diameter was measured using a Fiber Cashmere Fineness Analyzer (OFDA2000BT).

Genotypic Data: The genotypic data used in this study were derived from the GGP_Goat_70K SNP chip genotyping data of 2299 Inner Mongolian cashmere goats previously collected by the research team. Firstly, the individuals with recorded genotypes were extracted from a database of breeding information, and the outliers for each trait were further excluded. Values outside the range of the mean plus or minus 2.58 times the standard deviation were defined as outliers. Then, quality control of the genotype data was performed using PLINKv1.90 software. The quality control criteria were as follows: (1) exclude individuals with SNP missing rate of >10%; (2) exclude SNPs with individual missing rate of >10%; (3) exclude SNPs with minor allele frequency (MAF) of <5%; and (4) exclude SNPs violating the Hardy–Weinberg equilibrium (HWE) at *p* < 1 × 10^−5^. The missing genotypes were imputed using Beagle. Finally, a total of 50,728 SNPs in 2256 individuals remained for subsequent analyses.

### 2.2. Experimental Methods

#### 2.2.1. Environment Configuration

In this study, we employed various machine learning methods, including Random Forest (RF), Gradient Boosted Decision Tree (GBDT), XGBoost, and LightGBM, to predict phenotypes from genomic data. All prediction processes were implemented using the Python programming language and executed in the Jupyter Notebook 7.2.2 environment. Below is the detailed environment configuration: Operating System: Windows 11 64-bit; Python Version: Python 3.12.4; Jupyter Notebook Version: 7.2.2 and conda 24.9.2. Key Libraries and Frameworks: XGBoost 1.3.3: for implementing the XGBoost algorithm; LightGBM 4.6.0: for implementing the LightGBM algorithm; scikit-learn 1.5.1: for implementing the RF and GBDT algorithms; NumPy 1.26.4: for numerical computations; Pandas 2.2.2: for data processing and analysis; Matplotlib 3.9.2.

#### 2.2.2. Random Forest

Random Forest (RF) is a machine learning algorithm ensemble of numerous decision tree models, where predictions are collectively determined by multiple trees. First introduced by Breiman [18], RF comprises multiple decision trees. It estimates and fits numerous trees on diverse data subsets, leveraging their averages to enhance predictive accuracy and mitigate overfitting.

Random Forest algorithms can be expressed through mathematical formulas:

(1) $$F\left(x\right)=\frac{1}{B}\sum_{b=1}^{B}h_{b}(x)$$

where *F*(*x*) represents the predicted value of the random forest regression, *h_b_*(*x*) is the predicted result of the bth decision tree, and *B* denotes the number of decision trees in the random forest.

#### 2.2.3. Gradient Boosting Decision Tree

Gradient Boosting Decision Tree (GBDT) is a representative algorithm in boosting methods. It serves as the foundation for modern powerful algorithms like XGBoost and LightGBM (LGBM), and is among the most stable machine learning algorithms in practical applications. Initially introduced as Gradient Boosting Machine (GBM), it combines the ideas of Bagging and Boosting, leverages their advantages, accepts various types of weak learners as input, and was subsequently renamed Gradient Boosting Decision Tree (GBDT) after weak learners were primarily defined as decision trees. GBDT incorporates the three key elements of Boosting: loss function (*L*), weak learner (*F*(*x*)), and combined ensemble results (*F*(*x*)).

The Gradient Boosting Decision Tree algorithm can be represented mathematically as:

(2) $$F\left(x\right)=\sum_{m=1}^{M}\gamma_{m}h_{m}(x)$$

where *F*(*x*) represents the final prediction of the Gradient Boosting Decision Tree, *h_m_*(*x*) denotes the mth decision tree, *γ_m_* is the weight of the mth decision tree, and *M* represents the total number of decision trees.

#### 2.2.4. Extreme Gradient Boosting

Extreme Gradient Boosting (XGBoost) is a new-generation boosting algorithm based on the Gradient Boosting Decision Tree (GBDT). XGBoost is an algorithm system centered on boosting trees, designed to implement various types of gradient-boosted trees. Initially introduced by Chen Tianqi in 2014 [19], XGBoost was first presented in his doctoral dissertation. XGBoost is an ensemble learning algorithm that enhances the performance and generalization ability of decision tree models through the Gradient Boosting Framework.

The Extreme Gradient Boosting algorithm can be formulated as

(3) $$F\left(x\right)=\sum_{t=1}^{T}{f(x)}_{t}$$

where *F*(*x*) represents the final prediction of the Extreme Gradient Boosting tree, *f_t_*(*x*) is the predicted value of the *t*-th decision tree, and *T* is the total number of decision trees.

#### 2.2.5. Light Gradient Boosting Machine

Light Gradient Boosting Machine (LightGBM) is a highly efficient Gradient Boosting algorithm developed by the Microsoft Research Asia team. Initially designed for internal use at Microsoft to handle massive high-dimensional data, it was officially introduced in 2017 by Guolin Ke, Qi Meng, Thomas Finley, and others in a research paper [20]. LightGBM is a cutting-edge ensemble learning algorithm, similar to XGBoost, and both algorithms represent enhancements over GBDT. LightGBM offers superior computational efficiency and lower memory usage. Moreover, it demonstrates better resistance to overfitting when dealing with high-dimensional data, making it more suitable as a baseline model for preliminary exploratory modeling.

The Lightweight Gradient Boosting Machine algorithm can be formulated as

(4) $$F\left(x\right)=\sum_{t=1}^{T}\omega_{t}f_{t}(x)$$

where *F*(*x*) represents the final prediction of the Lightweight Gradient Boosting Machine, *f_t_*(*x*) is the predicted value of the *t*-th decision tree, and *T* is the total number of decision trees, and *ω_t_* is the weight of the *t*-th decision tree.

#### 2.2.6. GBLUP

(5) y=1μ+Zg+e

in which ***y*** is the observed trait. ***μ*** is the overall mean, **1** is a vector of 1 s, g is the vector of genomic breeding values, **e** is the vector of random errors, and Z is an incidence matrix allocating records to ***g***. The distributions of random effects were: ***g*** ~N(**0**, **G** *σ*^2^*_g_*) and e ~N(**0**, **I** *σ*^2^*_e_*), where **G** was the genomic relationship matrix (**G** matrix), and *σ*^2^*_g_* and *σ*^2^*_e_* were the additive genetic variance and the residual variance, respectively.

#### 2.2.7. Genomic Prediction Accuracy Evaluation

**Figure 1:**
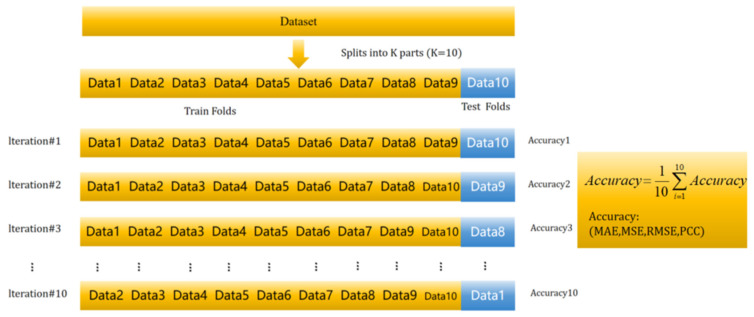
10-fold Cross-Validation.

In this study, we employed 10-fold cross-validation (10-fold CV) to evaluate the accuracy of genomic predictions. Specifically, the dataset was randomly partitioned into 10 subsets of approximately equal size, with nine used for model training and one for validation. This process was repeated 10 times, each time with a different subset reserved for validation, ensuring all samples were used for validation (Figure 1). This approach allowed us to comprehensively assess model performance across different data subsets, with cashmere producing a robust accuracy estimate.

The accuracy of genomic prediction was assessed by calculating the Pearson correlation coefficient between the corrected phenotypic values (yc) and the predicted values (pv). The Pearson correlation coefficient, ranging between −1 and 1, measures the linear relationship between two variables. A value closer to 1 or −1 indicates a stronger correlation, while a value near 0 suggests a weak correlation. A high Pearson correlation coefficient reflects a strong linear relationship between predicted and actual phenotypic values, indicating a more accurate model. To precisely evaluate model performance, we calculated the Pearson correlation coefficient between yc and pv for each validation set across the 10-fold cross-validation and averaged these coefficients to obtain the final accuracy metric for genomic prediction.

## 3. Results

### 3.1. Results of Descriptive Statistical Analysis of Phenotypic Data

The descriptive statistical analysis of phenotypic data is presented in Table 1, summarizing phenotypic traits (fiber length, cashmere diameter, and cashmere production) for 2256 Inner Mongolian cashmere goats (2011–2021). It includes the number of records, mean, standard deviation, coefficient of variation, and maximum and minimum values. Table 1 shows that the mean values for fiber length, cashmere diameter, and cashmere production were 18.90 cm, 15.23 µm, and 740.32 g, with coefficients of variation of 25.91%, 5.31%, and 29.07%, respectively. The cashmere diameter had a relatively low variation coefficient, while fiber length and cashmere production had higher ones, suggesting greater potential for improving these traits through selective breeding.

### 3.2. Optimal Hyperparameters of Machine Learning Models

This study used Bayes_opt optimization to tune the hyperparameters of RF, GBDT, XGBoost, and LightGBM, identifying the optimal hyperparameters for each machine learning model (Table 2). For RF, the maximum tree depth (max_depth) was set to 18, and the number of trees (n_estimators) to 250. For XGBoost, the number of trees (n_estimators) was 100, the learning rate (learning_rate) 0.1, and the maximum tree depth (max_depth) 5. For LightGBM, the learning rate (learning_rate) was 0.05, the maximum tree depth (max_depth) 5, and the number of leaves (num_leaves) 20. For GBDT, the number of trees (n_estimators) was 300, the learning rate (learning_rate) 0.1, and the maximum tree depth (max_depth) 5.

### 3.3. Genomic Selection Accuracy Evaluation

Genomic prediction accuracy for cashmere diameter, cashmere production, and fiber length in Inner Mongolian cashmere goats was evaluated using GBLUP, RF, GBDT, XGBoost, and LightGBM with 10-fold cross–validation. The accuracies of these machine learning methods are presented in Table 3. After optimizing the parameters of the four machine learning models using Bayesian optimization, the prediction accuracy exceeded that of the default parameter settings. Following hyperparameter tuning, the ML methods showed an average improvement in prediction accuracy of 2.9%, 2.7%, and 3.8% for cashmere diameter, cashmere production, and fiber length, respectively. Additionally, after hyperparameter adjustment, all machine learning methods except XGBoost showed higher accuracy than GBLUP. For fiber length, compared to GBLUP, RF showed a 0.3% improvement in prediction accuracy, LightGBM showed a 2.1% improvement, and GBDT showed a 1.4% improvement. After parameter optimization, LightGBM had the highest selection accuracy at 56.4%. For cashmere production, RF had the highest selection accuracy at 35.2% after parameter optimization. Compared to GBLUP, RF showed a 2.5% improvement in prediction accuracy, and LightGBM showed a 0.9% improvement. For cashmere diameter, after parameter optimization, GBDT had the highest selection accuracy at 40.4%. Compared to GBLUP, RF showed a 0.5% improvement, LightGBM showed a 0.6% improvement, and GBDT showed a 0.8% improvement. Furthermore, we found that the XGBoost algorithm had the lowest prediction accuracy for cashmere traits, with values of 0.541, 0.309, and 0.387, all of which were lower than those of GBLUP.

**Figure 2:**
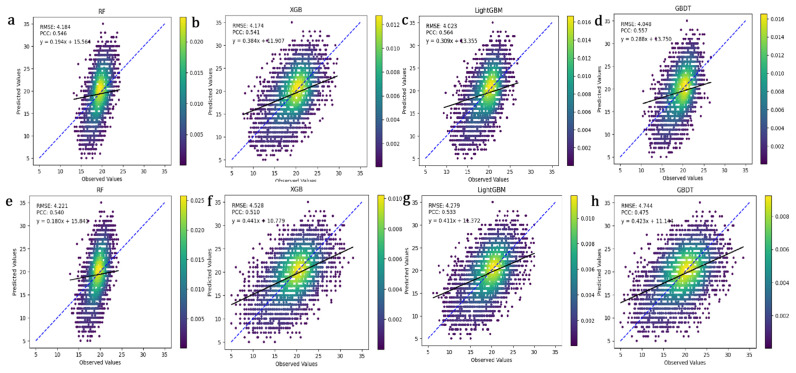
Impact of Four Machine Learning Methods on the Prediction Accuracy of Fiber Length Traits. The *X*-axis represents observed values, and the *Y*-axis represents predicted values. The points in the figure illustrate the relationship between model predictions and actual observations. The diagonal line indicates ideal predictions where predicted values equal observed values (Y = X line). The color bar on the right represents point density, with colors ranging from purple (low density) to yellow (high density). The blue line represents the ideal prediction scenario, where the predicted values perfectly align with the actual observed values. The black line is the linear regression line between the model predictions and the actual observations, showing the linear relationship between them. (**a**–**d**): Scatter plots of fiber length predictions vs. true values using RF, XGB, LightGBM, and GBDT with optimized hyperparameters. (**e**–**h**): Scatter plots of fiber length predictions vs. true values using RF, XGB, LightGBM, and GBDT with default hyperparameters.

**Figure 3:**
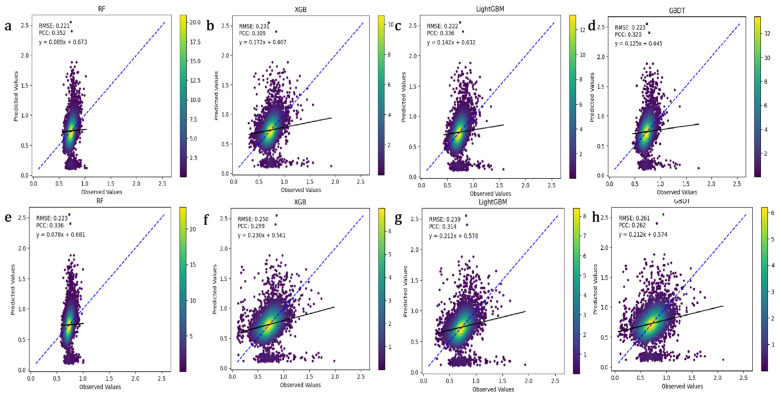
Impact of Four Machine Learning Algorithms on the Accuracy of Cashmere Production Traits. The blue line represents the ideal prediction scenario, where the predicted values perfectly align with the actual observed values. The black line is the linear regression line between the model predictions and the actual observations, showing the linear relationship between them. (**a**–**d**): Scatter plots of cashmere production predictions vs. true values using RF, XGB, LightGBM, and GBDT with optimized hyperparameters. (**e**–**h**): Scatter plots of cashmere production predictions vs. true values using RF, XGB, LightGBM, and GBDT with default hyperparameters.

**Figure 4:**
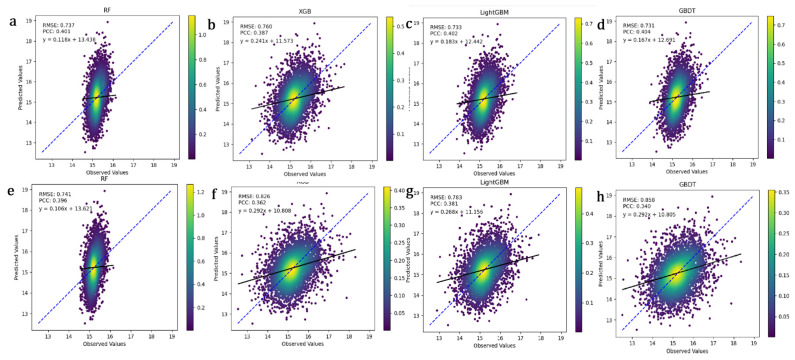
Impact of Four Machine Learning Algorithms on the Accuracy of Cashmere Diameter Traits. The blue line represents the ideal prediction scenario, where the predicted values perfectly align with the actual observed values. The black line is the linear regression line between the model predictions and the actual observations, showing the linear relationship between them. (**a**–**d**): Scatter plots of cashmere diameter predictions vs. true values using RF, XGB, LightGBM, and GBDT with optimized hyperparameters. (**e**–**h**): Scatter plots of cashmere diameter predictions vs. true values using RF, XGB, LightGBM, and GBDT with default hyperparameters.

This study compared the performance of RF, GBDT, XGBoost, and LightGBM in predicting fiber length, cashmere production, and cashmere diameter in Inner Mongolian cashmere goats. The Pearson correlation coefficient (PCC) measures the linear correlation between true and predicted values; a higher PCC indicates better model prediction accuracy. Root Mean Square Error (RMSE) quantifies the deviation between predicted and actual values; a lower RMSE signifies more accurate model predictions. As shown in Figure 2, LightGBM outperformed RF, GBDT, and XGBoost in predicting fiber length. It had the lowest RMSE of 4.023 and the highest PCC of 0.557 after optimization, indicating high prediction accuracy and stability. While XGBoost’s RMSE decreased by 8.0% after optimization, its overall performance remained slightly inferior to LightGBM. Overall, LightGBM demonstrated the best predictive performance. The study compared the prediction performance of RF, GBDT, XGBoost, and LightGBM for cashmere production (Figure 3). RF showed the best performance after optimization, with the lowest RMSE of 0.221 and the highest PCC of 0.352. After optimization, LightGBM’s RMSE decreased from 0.239 to 0.222, and its PCC increased from 0.314 to 0.336, showing significant improvement. The study also compared the prediction performance of the four models for cashmere diameter (Figure 4). GBDT performed the best after optimization, with the lowest RMSE of 0.731 and the highest PCC of 0.404, indicating high prediction accuracy and stability.

### 3.4. Evaluation of Error Metrics for Five Method

Mean Squared Error and Mean Absolute Error are commonly used to evaluate the performance of different machine learning methods. MSE considers both prediction accuracy and bias, while MAE better reflects the actual prediction error. Smaller MSE and MAE values indicate better model accuracy, lower error, and higher reliability. As shown in Table 4, after hyperparameter tuning, all machine learning methods exhibited reduced MSE and MAE values. After optimization, LightGBM had the smallest error for fiber length (MSE = 16.359, MAE = 3.239). GBDT had the smallest error for cashmere diameter (MSE = 0.533, MAE = 0.569). The three machine learning methods showed similar errors in predicting cashmere production. Compared to GBLUP, both LightGBM and GBDT exhibited lower MSE and MAE values. Meanwhile, when XGBoost is used to predict fiber length, cashmere production, and cashmere diameter, it yields the highest MSE and MAE values, indicating that it has the lowest prediction accuracy and the largest error magnitude for cashmere traits. With default hyperparameters, the Random Forest algorithm demonstrated the smallest errors for all three cashmere traits in Inner Mongolian cashmere goats.

### 3.5. Computational Efficiency of Four Machine Learning Algorithms

This study compared the average computing time per fold for different machine learning models during 10-fold cross-validation, with results shown in Table 5. Machine learning models with optimized hyperparameters generally outperformed those with default hyperparameters in terms of computing time. After hyperparameter optimization, GBDT had the lowest average computing time for all traits, at 95.51 s for cashmere production and 82.97 s for cashmere diameter. By contrast, XGB had the longest computing time for all traits. These results indicate that proper hyperparameter tuning can enhance model computational efficiency. Compared to the GBLUP method, the four machine learning algorithms demonstrated significantly higher efficiency, indicating that machine learning approaches can more effectively handle high-dimensional genomic data and construct nonlinear models.

## 4. Discussion

This study optimized the hyperparameters of RF, GBDT, XGBoost, and LightGBM using Bayes_opt, determining the best hyperparameter combinations for each machine learning method. After optimization, the average prediction accuracy for cashmere diameter, cashmere production, and fiber length improved by 2.9%, 2.7%, and 3.8%, respectively, highlighting the importance of hyperparameter tuning for ML algorithms. Liu et al. [21] compared seven ML methods (SVR, RF, GBDT, XGBoost, LightGBM, KRR, and MLP) for genomic selection accuracy in a yellow-feathered chicken population. After hyperparameter optimization, the accuracy of all seven ML methods improved, consistent with our findings. Wang et al. [22] evaluated genomic prediction accuracy in Chinese Yorkshire pigs using SVR, KRR, RF, and Adaboost.R2. The study found that RF’s prediction accuracy for total number born (TNB) and number born alive (NBA) improved by 9.8% and 10.2% after hyperparameter optimization, consistent with our study. This also indicates that hyperparameter tuning is essential for improving prediction accuracy in genomic selection using machine learning algorithms. In breeding practice, a model’s ability to accurately predict outcomes directly impacts the selection of superior individuals and the development of breeding strategies. Thus, hyperparameter tuning is not only a way to enhance model performance, but also a crucial step in achieving breeding objectives. Model optimization for improved prediction accuracy helps reliably identify individuals with superior cashmere traits, thereby guiding practical breeding decisions. This process ultimately improves breeding efficiency and economic outcomes by offering more precise support for breeding decisions.

Machine learning methods can flexibly capture potential nonlinear genotype-phenotype relationships and consider marker correlations and interactions. Consequently, in this study, RF, GBDT, XGBoost, and LightGBM were used with 10–fold cross–validation to evaluate the genomic prediction accuracy of cashmere diameter, cashmere production, and fiber length in Inner Mongolian cashmere goats (Albas type). The results showed that for fiber length, the selection accuracy of LightGBM achieved the highest value (56.4%) after parameter optimization, with the lowest MSE (16.359) and MAE (3.239). GBDT showed slightly lower accuracy compared to LightGBM in this trait. For cashmere production, RF demonstrated the highest selection accuracy at 35.2% after parameter optimization. Other models showed relatively lower performance in this trait. For cashmere diameter, GBDT achieved the highest selection accuracy (40.4%) after parameter optimization, with the minimum errors (MSE = 0.533 and MAE = 0.569). However, in the case of cashmere traits, the prediction accuracy of XGBoost was the lowest among all the models, at 0.541, 0.309, and 0.387 for fiber length, cashmere production, and cashmere diameter, respectively. Meanwhile, XGBoost produces the highest MSE and MAE values when predicting cashmere traits. This reflects that XGBoost achieves the lowest accuracy and exhibits the poorest performance in predicting cashmere traits. Wang et al. [23] compared eight ML algorithms and GBLUP for genomic selection accuracy in Yorkshire pigs and found that LightGBM consistently outperformed XGBoost in prediction accuracy for raw data, which is consistent with our study. Wang et al. [24] used six traditional and five ML methods to predict three reproductive traits (litter weight, total piglets born, and number of piglets born alive). The varying prediction accuracies of different models for distinct traits mainly result from the compatibility between model characteristics and trait features. While LightGBM, Random Forest, and Gradient Boosting Decision Trees all fall under the category of ensemble learning, they each have different focuses. In this study, the RF model showed the highest prediction accuracy for cashmere production. Biologically, this might be because cashmere production is primarily controlled by the additive effects of multiple genes and some non-additive effects. Random Forest introduces randomness through random sampling and feature selection, enabling it to better handle high-dimensional data and noise. For cashmere production traits, Random Forest can effectively capture both linear and nonlinear contributions of these genes. GBDT, on the other hand, optimizes step by step, focusing on the residuals of the previous iteration in each iteration, thus better capturing complex patterns and nonlinear relationships in the data. For fiber length and cashmere diameter traits, GBDT can more effectively model these complex genetic structures. This matching between model and trait characteristics enables different models to show varying accuracies in predicting different traits. Azodi et al. [25] found that no single GP algorithm performs best across all species and traits, which is consistent with our findings. They also found that ensemble-based predictions are more stable and likely to yield better accuracy than single algorithms. Thus, for predicting cashmere traits in cashmere goats, we can consider ensemble predictions that leverage the capabilities of multiple algorithms.

Furthermore, after optimizing the hyperparameters, all machine learning methods showed reductions in both MSE and MAE. With the optimized hyperparameters, LightGBM demonstrated the smallest error for fiber length, and GBDT for cashmere diameter. The three machine learning methods showed similar errors in predicting cashmere production. When using the default hyperparameters, the Random Forest algorithm showed the smallest errors for all three cashmere traits in Inner Mongolian cashmere goats. Zhang et al. [26] assessed the genomic prediction accuracy of reproductive traits in 385 Large White pigs using GBDT, RF, LightGBM, Adaboost.R2, GBLUP, BRR, and BL. The results highlighted the critical role of hyperparameter tuning in optimizing machine learning models and showed that the LightGBM model outperformed RF in both MSE and RMSE, which is consistent with our findings. This study also explored the average computing time per fold for different machine learning models during 10-fold cross-validation. Results indicated that XGB had the longest computing time for all traits. Compared with the GBLUP method, the four machine learning algorithms showed significantly higher efficiency, indicating that machine learning approaches can more effectively handle high–dimensional genomic data and build nonlinear models. However, Chen et al. [27] found that in predicting pig growth traits and GEBV, RF had the longest training time, while XGB had the shortest, contradicting our results. These inconsistencies might arise from differences in species, data size, and computer configuration.

Compared with GBLUP, machine learning algorithms show advantages in handling complex genetic data, such as capturing nonlinear relationships and high-dimensional features. For fiber length, compared with GBLUP, RF improved prediction accuracy by 0.3%, LightGBM by 2.1%, and GBDT by 1.4%; for cashmere production, RF by 2.5%, LightGBM by 0.9%; and for cashmere diameter, RF by 0.5%, LightGBM by 0.6%, and GBDT by 0.8%. This indicates that machine learning algorithms show higher accuracy in predicting complex traits, especially those with low heritability or involving multiple gene interactions. This means machine learning algorithms can more comprehensively utilize genetic information and enhance the interpretation of genetic variations. Furthermore, machine learning algorithms can identify genetic markers more related to target traits, improving genomic prediction accuracy, and subsequently optimizing parent selection and offspring prediction. Additionally, compared with the GBLUP method, the four machine learning algorithms showed significantly higher efficiency, indicating that machine learning approaches can more effectively handle high-dimensional genomic data and build nonlinear models. The efficiency and flexibility of machine learning algorithms help reduce the cost and time of genomic selection, making breeding programs more cost-effective. Specifically, machine learning algorithms can be integrated into genomic selection programs. By customizing model selection and parameter tuning, they can achieve precise prediction and optimization for different traits and breeding objectives. This not only improves breeding efficiency but also enables early selection and reduces generation intervals.

This study has certain limitations, including a moderate sample size, potential inability to capture small–effect SNPs, and limited interpretability of machine learning models. Machine learning is an efficient data processing method. Machine learning can effectively handle high–dimensional genomic data and build nonlinear models for more accurate genomic predictions. As the reference population size increases, machine learning methods can capture more information during model training and produce more accurate predictions. This study used 70KSNP chip data for genomic predictions. It is worth considering whether using low–depth resequencing data alongside chip data could provide more training information and improve accuracy. Additionally, genome–wide association studies (GWAS) can identify genetic variants related to multiple traits. Integrating GWAS into GS models and incorporating trait–specific markers can enhance genomic predictions [28,29]. Utilizing GWAS results to enhance GS models and improve the prediction accuracy of related traits offers an alternative method to boost the biological interpretability of GS models.

## 5. Conclusions

In this study, we conducted genomic prediction for Inner Mongolian cashmere goats using GBLUP, RF, XGBoost, GBDT, and LightGBM, and compared the prediction accuracy, mean squared error (MSE), mean absolute error (MAE), and computing time of these methods. The results showed that for fiber length, LightGBM had the highest selection accuracy of 56.4%; for cashmere production, RF had the highest selection accuracy of 35.2%; and for cashmere diameter, GBDT had the highest selection accuracy of 40.4%. Among the three traits, XGBoost had the lowest prediction accuracy, at 0.541, 0.309, and 0.387 for fiber length, cashmere production, and cashmere diameter. Additionally, our results indicate that hyperparameter tuning is necessary when using machine learning algorithms for genome-wide selection, as it can improve prediction accuracy.

## Figures and Tables

**Table 1:** Summary of Basic Statistical Summary of Traits.

Trait	Number of Record	Mean	SD	Coefficient of Variation
FL/cm ^1^	9610	18.90	4.90	25.91%
CD/µm ^2^	5356	15.23 ^1^	0.81	5.31%
CP/g ^3^	9040	740.32	215.19	29.07%

^1^ FL: fiber length; ^2^ CD: cashmere diameter; ^3^ CP: cashmere production.

**Table 2:** Optimal hyperparameters for each machine learning algorithm.

Method	Optimal Hyperparameters
RF	max_depth = 18, n_estimators = 250
XGBoost	n_estimators = 100, learning_rate = 0.1, max_depth = 5
LightGBM	learning_rate = 0.05, max_depth = 5, num_leaves = 20
GBDT	n_estimators = 300, learning_rate = 0.1, max_depth = 5

**Table 3:** Predictive accuracy of five methods for three traits using 10-fold cross-validation.

Hyperparameters	Method	FL ^1^	CP ^2^	CD ^3^
Tuning ^4^	GBLUP	0.543 ± 0.0315	0.327 ± 0.0478	0.396 ± 0.0361
	RF	0.546 ± 0.0699	0.352 ± 0.0397	0.401 ± 0.0397
	XGB	0.541 ± 0.0475	0.309 ± 0.0794	0.387 ± 0.0325
	LightGBM	0.564 ± 0.0129	0.336 ± 0.0799	0.402 ± 0.0359
	GBDT	0.557 ± 0.0423	0.320 ± 0.0631	0.404 ± 0.0310
Default ^5^	RF	0.540 ± 0.0509	0.336 ± 0.0699	0.396 ± 0.0354
	XGB	0.510 ± 0.0443	0.299 ± 0.0738	0.362 ± 0.0347
	LightGBM	0.533 ± 0.0445	0.314 ± 0.0755	0.381 ± 0.0330
	GBDT	0.475 ± 0.0478	0.262 ± 0.0678	0.340 ± 0.0388

^1^ FL: fiber length; ^2^ CP: cashmere production; ^3^ CD: cashmere diameter; Tuning ^4^: hyperparameter optimization; Default ^5^: default hyperparameters.

**Table 4:** Five methods’ mean squared error and mean absolute error.

Hyperparameters	Method	FL ^1^		CP ^2^		CD ^3^	
		MSE ^4^/cm^2^	MAE ^5^/cm^2^	MSE/g^2^	MAE/g^2^	MSE/µm^2^	MAE/µm^2^
	GBLUP	16.716	3.267	0.050	0.149	0.565	0.576
Tuning	RF	17.508	3.343	0.049	0.150	0.543	0.574
	XGBoost	17.419	3.340	0.053	0.154	0.578	0.591
	LightGBM	16.359	3.239	0.049	0.149	0.537	0.570
	GBDT	16.384	3.252	0.050	0.150	0.533	0.569
Default	RF	17.814	3.370	0.050	0.152	0.549	0.578
	XGBoost	20.503	3.610	0.063	0.170	0.682	0.637
	LightGBM	18.306	3.426	0.057	0.160	0.613	0.605
	GBDT	22.502	3.769	0.068	0.179	0.736	0.662

^1^ FL: fiber length; ^2^ CP: cashmere production; ^3^ CD: cashmere diameter; ^4^ MSE: Mean Squared Error; ^5^ MAE: Mean Absolute Error.

**Table 5:** Average computing time to complete each fold of 10-fold CV according to different genomic prediction methods.

Hyper-Parameters	Method	FL ^1^	CP ^2^	CD ^3^
	GBLUP	9748.25 s	19,351.12 s	62,875.9 s
Tuning	RF	131.45 s	128.14 s	139.05 s
	XGB	421.79 s	433.66 s	382.62 s
	LightGBM	199.41 s	233.12 s	222.49 s
	GBDT	107.23 s	95.51 s	82.97 s
Default	RF	189.47 s	135.45 s	189.47 s
	XGB	482.26 s	462.17 s	401.31 s
	LightGBM	247.33 s	282.89 s	235.86 s
	GBDT	200.77 s	106.18 s	86.29 s

^1^ FL: fiber length; ^2^ CP: cashmere production; ^3^ CD: cashmere diameter.

## Data Availability

The data that support the findings of this study are available from the corresponding authors upon reasonable request.
